# High-resolution synchrotron-based X-ray microtomography as a tool to unveil the three-dimensional neuronal architecture of the brain

**DOI:** 10.1038/s41598-018-30501-x

**Published:** 2018-08-13

**Authors:** Matheus de Castro Fonseca, Bruno Henrique Silva Araujo, Carlos Sato Baraldi Dias, Nathaly Lopes Archilha, Dionísio Pedro Amorim Neto, Esper Cavalheiro, Harry Westfahl, Antônio José Roque da Silva, Kleber Gomes Franchini

**Affiliations:** 1 Brazilian Biosciences National Laboratory (LNBio), Brazilian Center for Research in Energy and Materials (CNPEM), Zip Code 13083-970 Campinas, Sao Paulo Brazil; 2 Brazilian Synchrotron Light National Laboratory (LNLS), Brazilian Center for Research in Energy and Materials (CNPEM), Zip Code 13083-970 Campinas, Sao Paulo Brazil; 3 Department of Neurology and Neurosurgery, Federal University of São Paulo (UNIFESP/EPM), Zip Code 04021-001 São Paulo, São Paulo Brazil

## Abstract

The assessment of neuronal number, spatial organization and connectivity is fundamental for a complete understanding of brain function. However, the evaluation of the three-dimensional (3D) brain cytoarchitecture at cellular resolution persists as a great challenge in the field of neuroscience. In this context, X-ray microtomography has shown to be a valuable non-destructive tool for imaging a broad range of samples, from dense materials to soft biological specimens, arisen as a new method for deciphering the cytoarchitecture and connectivity of the brain. In this work we present a method for imaging whole neurons in the brain, combining synchrotron-based X-ray microtomography with the Golgi-Cox mercury-based impregnation protocol. In contrast to optical 3D techniques, the approach shown here does neither require tissue slicing or clearing, and allows the investigation of several cells within a 3D region of the brain.

## Introduction

Neurons have distinct morphology and specific patterns of connectivity, which are essential for its proper functioning. The neuronal organization and network are the basis for understanding brain function in health and disease. Therefore, the deep comprehension of these features may help to uncover the whole functioning of the brain as a unit, based on the individualized activity of its main components, the neurons^1^.

The study of neurons as individualized unities has its beginning in the 19^th^ century with the creation of the Neuron Doctrine, established as a result of the work developed by Santiago Ramon y Cajal on the basis of histological techniques developed by Camillo Golgi^2–4^. Since then, neuroscientists have been pursuing enhanced ways to improve the view of brain cells and networks. However, the assessment of the three-dimensional (3D) brain cytoarchitecture with a sub-cellular resolution is still one of the greatest challenges in neuroscience. In the past two decades, a great variety of new techniques has been developed to explore the neural circuits of the whole brain^5–9^. Nevertheless, confocal optical microscopy is still the main method for 3D visualization of brain cells at a microscopic level^10^. In addition, well established methods such as magnetic resonance imaging^11^, serial block-face electron microscopy^12^ and histological sectioning^13^, although also commonly used, still face some issues such as lack of contrast, extensive sample preparation protocols and the destructive nature of serial sectioning. Therefore, new 3D imaging techniques are needed to assess increasingly larger brain volumes architecture at single cell level.

Since its discovery, X-rays showed to be an important non-destructive tool for imaging a broad range of samples, from dense materials to soft biological specimens. In addition, in the last few years, X-ray computed microtomography (X-ray μCT) appeared as a new method for deciphering the cytoarchitecture and connectivity of the brain in a non-destructive manner due to its high penetration depth, providing 3D information of different biological structures^14,15^. The tomographic slices are digitally reconstructed from several projection images acquired by a rotational scan, a very straightforward process compared with serial sectioning^16,17^. These projection images can be formed by numerous contrast mechanisms, based on the different X-ray‒matter interaction cross sections. Absorption contrast imaging, based on inhomogeneous transmission of the X-ray beam through the mass density distribution of a sample, was the first and possibly yet the most widespread form of X-ray imaging. Nonetheless, the absorption cross-sections of the lighter elements that constitute soft biological tissues, like the brain, are small, resulting in a weak absorption contrast.

Other contrast methods have to be employed in order to isolate structures within a brain sample. For instance, the inhomogeneous phase shifts experienced by the X-ray wave field traversing an object can be a more efficient contrast mechanism. After free space propagation, these phase shifts result into intensity modulations that can be orders of magnitude larger than the ones caused by the inhomogeneous absorption. This so-called propagation-based X-ray phase-contrast method has been recently exploited to reveal single-cell resolution images of a mouse brain cytoarchitecture^18^ and also to resolve the structure of entire myelinated mouse nerves^19^. Another efficient way of increasing the X-ray imaging contrast is by impregnating the biological sample with a chemical compound that has a higher-Z element in its composition. These staining-based methods require more elaborate sample preparation but, by exploring the selective affinity of these molecules with parts of the soft biological tissue, can be used to discriminate more specific brain structures^16–18,20,21^. However, due to the intrinsic ultra-high connectivity of the neuronal network, segmentation of whole neurons, *i.e*. partitioning the brain tomographic data into non-overlapping regions that represent the neuron cell body, axon and dendritic structure, was not possible so far. Not only the segmentation of entire neurons becomes inaccurate, but also extremely cumbersome in these highly connected and densely packed brain tissue. Consequently, the segmentation of a subset of more sparsely isolated whole neurons in 3D brain images, though lacking the complete neuronal connectivity information, can still provide evidence for the morphological and spatial organization of the brain neuronal network. This information can potentially reveal invaluable clues about the mechanisms underlying both normal brain function and pathology.

The Golgi-Cox staining method^22,23^ is a powerful technique to study neuronal connection, morphology as well as glial components. This method is commonly employed in histological techniques, since it is capable to stain only a small fraction of whole neuronal elements (of the order of 3–10%), allowing segmentation of single neurons for long distances in 2D optical images. Despite the fact that only a small fraction of neurons is impregnated with the stain, cells that are rendered more visible by the staining agent and maintain all features including cell body, dendrites, dendritic spines and axons.

Here we present a method of imaging combining synchrotron-based X-ray microtomography with the Golgi-Cox impregnation protocol^22,23^, that offers a higher and more homogeneous contrast of relatively sparsely distributed whole neurons within the tissue. In contrast to other optical 3D techniques, the approach shown here does neither require tissue slicing or clearing, then allowing the investigation of several cells within a 3D region of a brain structure.

## Results

### Brain cell distribution and organization using Golgi-Cox staining

The assessment of the three-dimensional cytoarchitecture and neuronal network has an enormous potential to the mechanisms underlying brain functioning and to lead to the development of advanced and targeted treatments.

**Figure 1:**
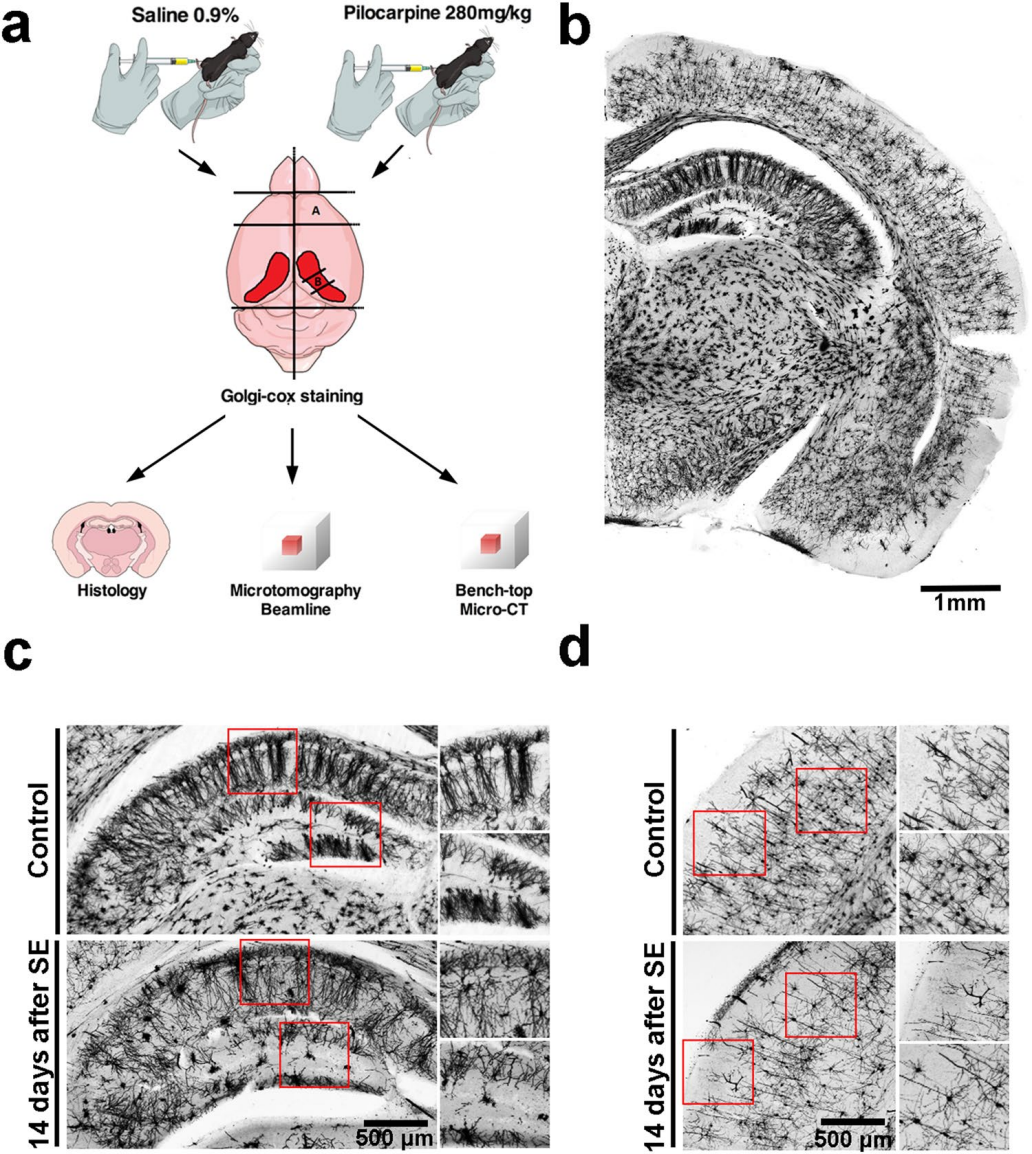
A schematic illustration of the experimental procedures. (**a**) Adult mice were i.p. injected with saline or pilocarpine solution (280 mg/kg). After pilocarpine injection the animals were continuously monitored over 24 h. Following 14 days of SE, the brains were removed and frontal cortex and medial part of the hippocampus were dissected. Briefly, brain structures were immersed into Golgi-Cox solution, where remained for 14 days. After, structures were washed, dehydrated and embedded in paraffin. At least 4 of each brain structure (cortex or hippocampus) were imaged in optical microscope, synchrotron-based microtomography beamline or on a bench-top μCT. Cartoons are courtesy of www.mindthegraph.com (mouse i.p injection and mouse brain). (**b**) A brain hemisphere showing the neurons in several regions of the sliced brain evenly and reliably stained. (**c**) Magnified images of Golgi-Cox labeled cerebral cortex from healthy (upper panels and inserts) or pilocarpine-treated animals (lower panel and inserts). (**d**) Magnified images of Golgi-Cox labeled hippocampus from healthy (upper panels and inserts) or pilocarpine-treated animals (lower panel and inserts).

To demonstrate that the Golgi-Cox staining offers good contrast for X-ray imaging and enables the study of the *in situ* neuronal cytoarchitecture and 3D-organization, we evaluated the distribution of neurons in the brains of control mice compared to a well-characterized model of brain damage based on the pilocarpine-induced *Status Epilepticus* (SE), a period of time comprehended by seizures lasting longer than 30 min, which leads to severe and widespread cell loss in several brain areas^24^. Figure 1a represents the experimental protocol used for this work. After Golgi-Cox impregnation, whole brains or isolated structures (frontal cortex and medium part of the hippocampus) were submitted to different imaging methods: conventional histology, synchrotron-based X-ray μCT or bench-top μCT. As observed in an 150μm-thick coronal slice of the control brain hemisphere (Fig. 1b), neurons are evenly and reliably stained in all brain areas. While Golgi-Cox impregnation was evident throughout all brain cortex and hippocampal regions of the control group slices, the qualitative analysis of the pilocarpine-treated group 14 days after *SE*, clearly shows a reduction of cell number and altered distribution of neurons specially in the hippocampus (Fig. 1c), but also in the frontal brain cortex (Fig. 1d).

### Synchrotron-based X-ray imaging

**Figure 2:**
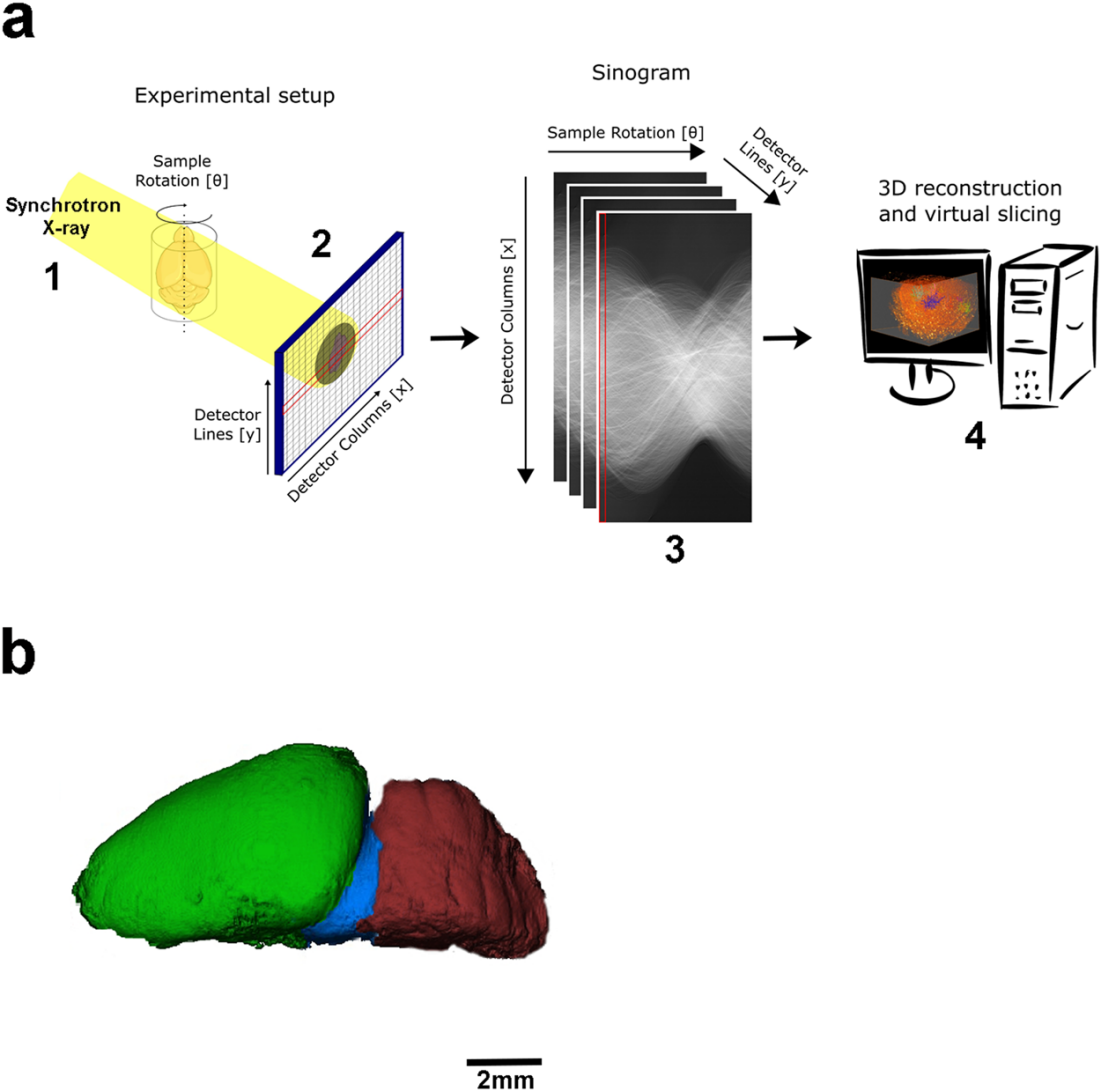
Experimental setup for the structural tomographic imaging of the nervous system. (**a**) Schematic view of the experimental setup for synchrotron-based X-ray microtomography. (1) X-rays from a synchrotron bending magnet source illuminated the samples positioned in front of an indirect X-ray detector (2). (3) Several projection images were acquired by a rotational scan and used to digitally reconstruct the tomographic slices (4). (**b**) Segmentation of one mouse brain hemisphere, showing the brain cortex (green), midbrain (blue) and cerebellum (red).

Since Golgi silver or mercury impregnation is a conventional sample preparation for the optical observation of neural cells, and capable to stain only a small fraction of whole neuronal elements, we decided to explore the benefits of this histological technique for 3D imaging using synchrotron-base X-ray microtomography. Figure 2a portrays the overview of the experimental setup used at the synchrotron (UVX-LNLS, Brazil) beamline for X-ray microtomography (IMX). The sample was positioned at 40 mm away from the detector which had an adjustable pixel size of 0.82 μm for high resolution/small FOV imaging and 4.11 μm for low resolution/large FOV imaging. All the details of image acquisition and setup are better explained in the methodology section. At first, a whole control mouse brain was imaged at the beamline and one hemisphere was digitally segmented, highlighting the brain cortex (green), midbrain (blue) and cerebellum (red) (Fig. 2b).

In order to acquire good tomographic data, the sample usually should be smaller than the field of view (FOV). At the same time, for a desired magnification, the FOV will be proportional to the pixel size, resulting on a compromise between sample size and magnification. In this work, we performed both low and high-resolution acquisitions for various FOV. Due to the limitation regarding the FOV that limited the sample size, as mentioned in the methodology section, and with the aim to go deeper into the 3D observation of the architecture of the brain neurons, tissues were dissected and two distinct regions were selected for further imaging. The frontal cortex and medium region of the hippocampus were the structures of interest due to the feasibility of reproducing the dissection process in different animals.

### X-ray tomography for neuromorphology and neuropathologic evaluation

**Figure 3:**
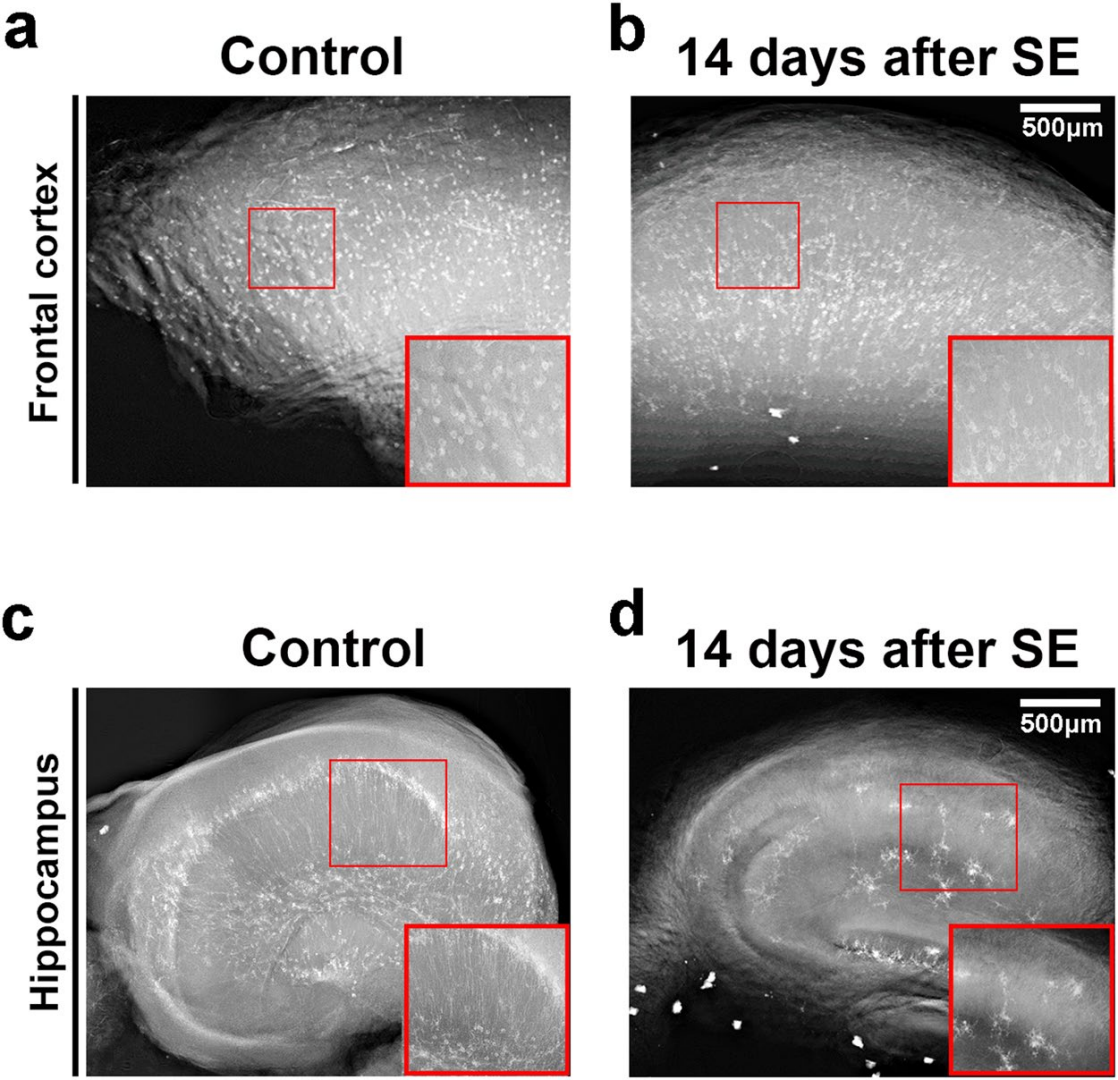
Representative X-ray absorption projections of Golgi-Cox labeled brain structures. X-ray projection of a hemi-frontal cortex from control (**a**) or pilocarpine-treated animals. (**b**) X-ray projection of the medium part of a hippocampus from control (**c**) or pilocarpine-treated animals (**d**). Regions of interest show in detail the mercury-impregnated neurons with typical cell body and neurites. Inserts from (**c**) and (**d**) show a significant reduction on total number of hippocampal neurons together with an alteration on cell branching and distribution.

**Figure 4:**
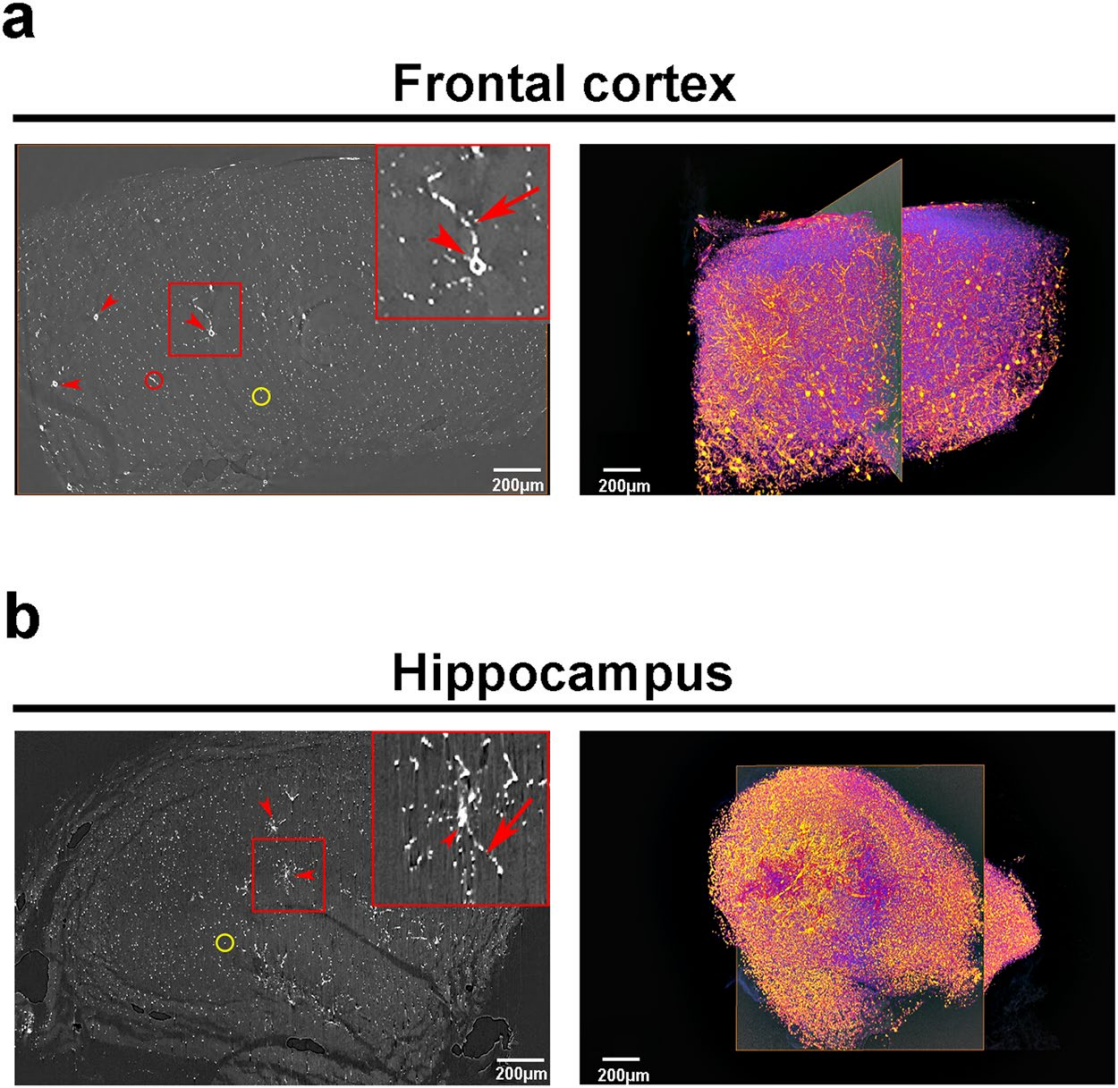
Synchrotron X-ray microtomography of mercury-impregnated neurons allows virtual slicing and 3D rendering of whole brain structures. Reconstructed slice (left panel) and 3D image rendering (right panel) of a control frontal cortex (**a**) and hippocampus (**b**). Rectangles in (**a**) and (**b**) highlights, respectively, a single cortical or hippocampal neuron. Arrowheads: neuron cell bodies; arrows: neurites; red circles: longitudinal virtual slicing of a neurite; yellow circles: transversal virtual slicing of a neurite.

**Figure 5:**
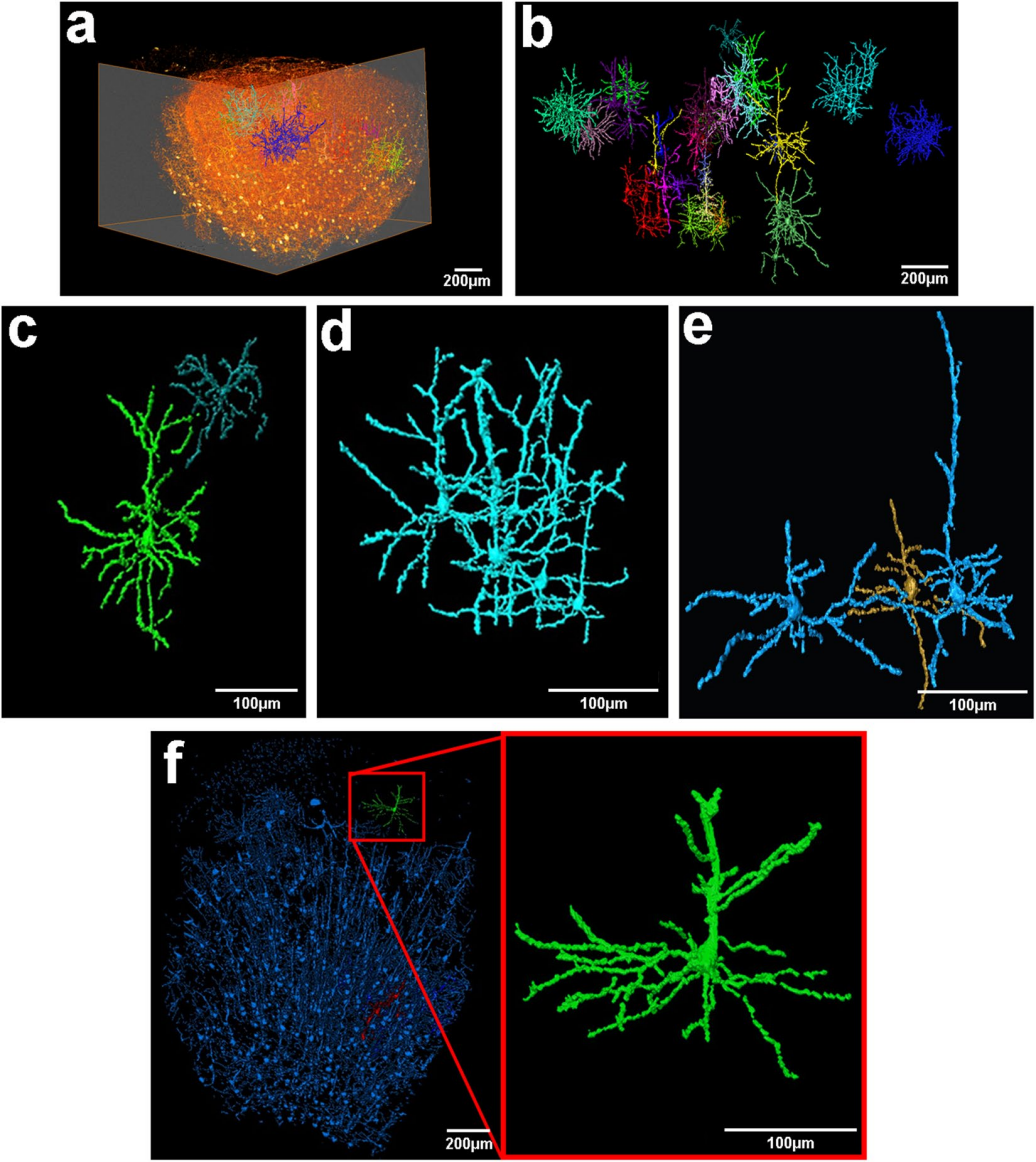
3D volume rendering down to cellular level of brain cortical neurons. (**a**) Volume rendering of a whole hemi-frontal cortex showing some manually segmented neurons within the structure. (**b**) Cellular segmentation of some cortical neurons of the structure represented in (**a**). Colors highlight a single neuron or a cluster of cells that could not be separated due to close proximity. (**c,d,e**) Manual segmentation of cortical neurons shown in (**b**). Missing connections of the neurites might be due to incomplete staining. The technique allows the characterization of morphologically distinct neurons. In (**c**) is represented a pyramidal neuron in green and a typical cortical interneuron (dark blue). Due to close proximity, a cluster of pyramidal cells are represented in (**d**) as a same connected object. Same pattern is represented in (**e**) for the two pyramidal neurons shown in blue. (**f**) Semiautomatic segmentation of frontal cortex (blue) based on the establishment of a gray-value threshold. Observe that this process does not provide much detail of the cells. Only after manual segmentation (green neuron, insert), all cell features and neurites are perfectly detailed. Note that missing connections of the neurites from the neuron highlighted in green might be due to incomplete staining. The data were from frontal brain cortex with a pixel size of ~820 nm.

**Figure 6:**
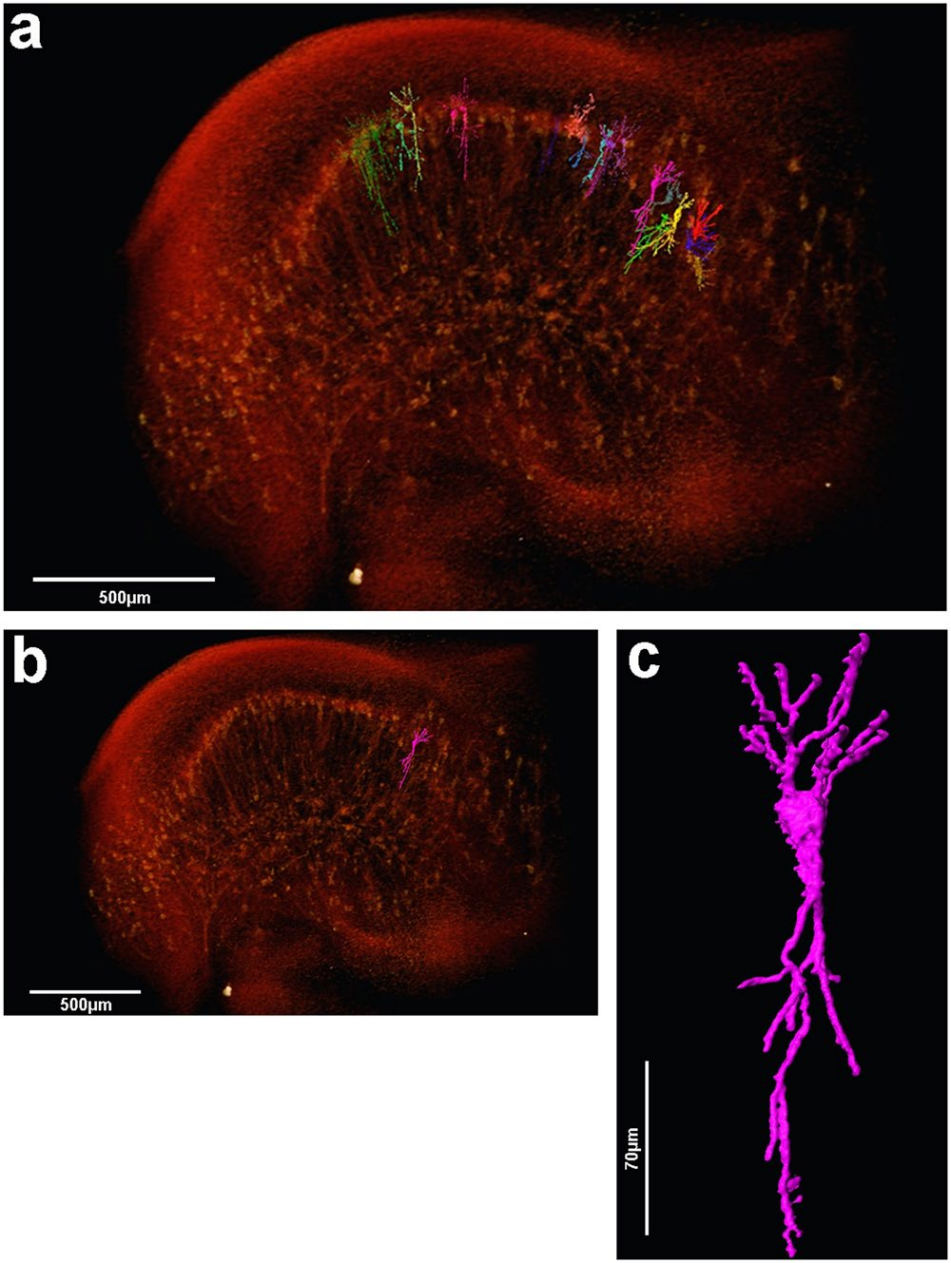
3D volume rendering and manual cell segmentation of µCT data from the mouse hippocampus. (**a**) Volume rendering of the complete medium part of the hippocampus showing some manually segmented pyramidal neurons from CA1 and CA3 regions. (**b**) Same image shown on (**a**) now highlighting only one pyramidal neuron from CA3 region. The same cell is amplified in (**c**) to allow the observation of the detailed morphology of the cell in a 3D perspective.

The hippocampus is one of the most affected structures due to pilocarpine administration, and was thus chosen for a comparative analysis^24–26^. Representative X-ray absorption projections of Golgi-Cox labeled brain structures are shown in Fig. 3. From these flat field-corrected projections, the contrast exhibited by the mercury impregnation is clearly observed, allowing a fair comparison between the two experimental groups. Brighter structures represent cell bodies and neurites from neurons that were mercury-labeled during the impregnation process. Pilocarpine-treated animals, as already described, presented a significant decrement in cell number in both structures analyzed (Fig. 3a,b, frontal cortex and Fig. 3c,d, hippocampus). After obtaining all projections, the images can be virtually sliced and 3D rendered, allowing the visualization of the structure as a whole. In Fig. 4a, a virtual slice (left panel) and a volume rendering (right panel) from the frontal cortex of a healthy animal are presented. It is possible to observe the soma and the neurites of several neuron in the reconstructed slice, after applying a threshold to highlight the mercury-impregnated cells. However, the thickness of certain regions of cell neurites were too close to the spatial resolution limit, blurring cell limits during automatic segmentation. The same was observed for the hippocampus of healthy animals (Fig. 4b). Therefore, we had to take hand of a manual segmentation to precisely analyze the morphology of single neurons. When we follow all the neuronal branching reaching a cell body, along the Z-series of projections, it is possible to perform a complete 3D reconstruction of the whole cell (see Supplementary Movies S1 and S2). Figure 5a shows a volume rendering of a whole hemi-frontal cortex highlighting in distinct colors some manually segmented neurons within the structure. Manual segmentation results in tremendously rich, detailed structures (Fig. 5b). For example, in Fig. 5c, two different cortical cell types are represented (a pyramidal cell and a cortical interneuron). However, the spatial resolution provided by the IMX beamline setup still did not allow the separation of structures closer than 1 μm in the vertical direction and 3 μm in the horizontal direction due to the penumbra effect caused by the X-ray source size in this 2^nd^ generation storage ring. Therefore, due to this close proximity, some neurons are represented in the same color, highlighting a cluster of cells that could not be separated (Fig. 5d,e). Figure 5f portrays the critical difference between the manual and the semi-automatic segmentation of the neurons. Neurons segmented with the semi-automatic approach based on the establishment of a gray-value threshold are shown in blue. Observe that this process does not provide much detail of the cells. Only after manual segmentation (green neuron, insert), the entire cell features, including neurites, are detailed. Some missing connections of the neurites from the neuron highlighted in green are possibly due to incomplete staining, it did not harm the manual tracing process. Manual segmentation was applied to hippocampus and the result is shown in Fig. 6a–c.

**Figure 7:**
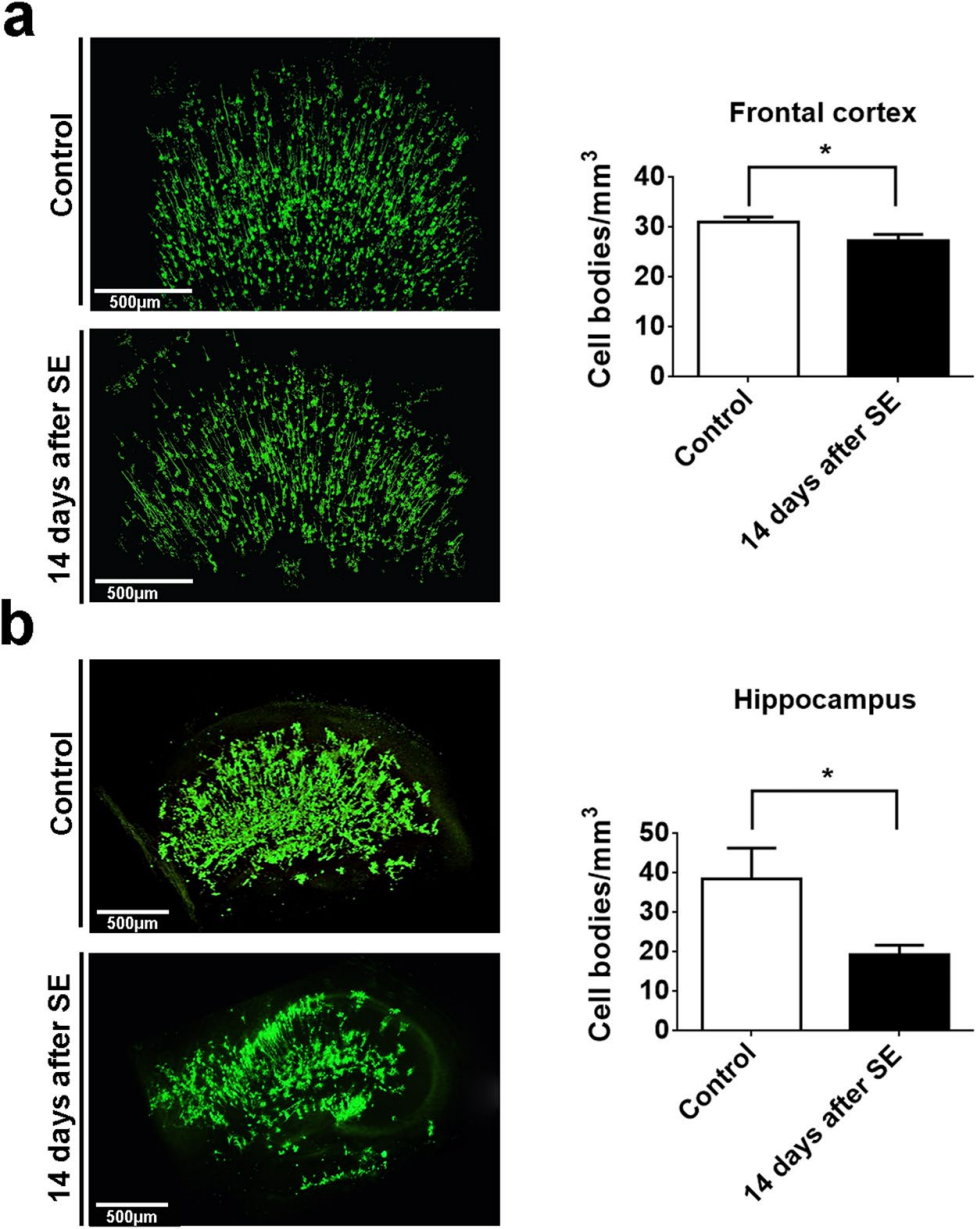
3D cell number quantification of mercury-impregnated neurons from frontal cortex and hippocampus of control and pilocarpine-treated mice. (**a**) Representative frontal cortex image of neuron mercury-impregnated in control and pilocarpine treated mice. Bar graph shows the significant difference in frontal cortex cell number quantification between control and pilocarpine-treated mice (Control: 31.08 ± 0.5678 *vs* 14-days after SE: 27.32 ± 0.9050; n = 5). (**b**) Representative hippocampal formation image of neurons mercury-impregnated in control and pilocarpine treated mice. Bar graph shows the significant difference in hippocampus cell number quantification between control and pilocarpine-treated mice (Control: 38.51 ± 5.5 *vs* 14-days after SE: 19.32 ± 1.6; n = 5). *p < 0.05

Next, we analyzed the damage induced by SE in the frontal cortex and the medial part of the hippocampus. As expected, we observed a significant decrease in cell density in frontal cortex (Control: 31.08 ± 0.5678 cells/mm^3^ *vs* 14-days after SE: 27.32 ± 0.9050 cells/mm^3^; n = 5; p < 0.05) (Fig. 7a) and in the hippocampal formation (Control: 38.51 ± 5.5 cells/mm^3^ *vs* 14-days after SE: 19.32 ± 1.6 cells/mm^3^; n = 5; p < 0.05) (Fig. 7b) in pilocarpine-treated animals, when compared to control group. In addition, qualitative analyses of hippocampus revealed an extensive loss of mercury-impregnated neurons in the granular layer of dentate gyrus, CA3 and CA1 subfields. The details regarding cell quantification are explained in the data processing section.

**Figure 8:**
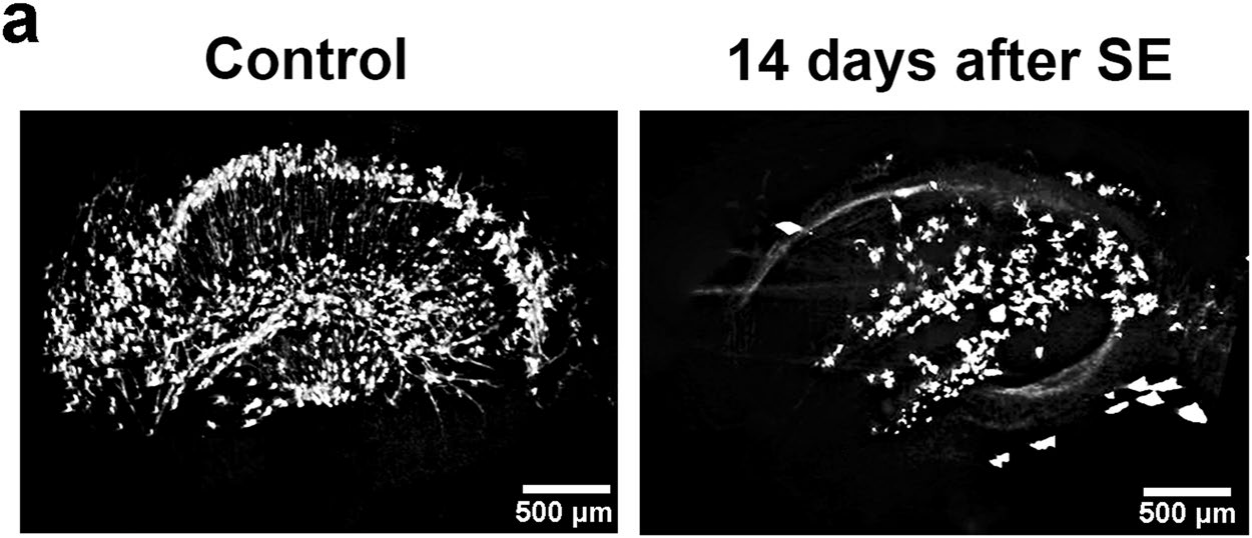
Bench-top X-ray microtomography reconstruction of mercury-impregnated hippocampus. (**a**) Preliminary tests done on a bench-top SKYSCAN 1272. Although a qualitative analysis may allow distinguishing between control and pilorcapine-treated animals a quantitative analysis was not possible due to the pixel size offered by the equipment.

In order to evaluate the Golgi-Cox labeling procedure on a bench-top μCT we also conducted experiments on a Brucker Skyscan 1272 μCT scanner using the same sample preparation procedure described previously. The resolution achieved with this system was not as high as the one obtained at the IMX beamline thus allowing only a qualitative analysis. Nevertheless, the striking difference between control and pilocarpine-treated groups is well observed, demonstrating the versatility of the procedure presented at this work (Fig. 8). Furthermore, we anticipate that the use of higher resolution bench-top μCT could allow the usage of the protocol for a quantitative analysis similar to the one presented here, allowing for a broader use and incorporation on a medical facility.

Therefore, Golgi-Cox labeling technique allied to X-ray synchrotron imaging revealed to be a powerful tool to analyze *in situ* and three dimensionally the neuronal morphology and distribution.

## Discussion

In the last few years, great efforts have been made in order to deeply understand neuronal organization and connectivity in intact brain samples. A promising approach to do that has been the usage of X-ray μCT, which appeared as a new method for deciphering the cytoarchitecture and connectivity of the brain in a non-destructive manner, providing 3D information of several biological structures^14,15^. A recent work, for example, showed that even unstained brain samples can be imaged under conventional bench-top X-ray μCT^18^. However, specific staining is still very desirable to study cell morphology, once it makes more feasible to isolate single cells in a mass of brain matrix.

The Golgi-Cox labeling technique offers suitable contrast for the specific labeling of neurons for X-ray microtomography, and the greatest potential of synchrotron-based X-ray microtomography lies in the ability of offering 3D visualization of the imaged samples.

Previous works have already demonstrated the applicability of μCT technique for 3D imaging of metal-based neuron impregnation. These works have used the Golgi labeling protocol, based on silver impregnation, and were able to reveal the microarchitecture of the gray and white matter of the human brain^16^. Other works from the same group have also showed the 3D network of the *Drosophila* brain hemisphere, detailing possible contact sites^27–29^.

So far, however, visualization of single cell morphology of intact neuronal tissues with X-ray μCT have not been completely defined due to a partially heterogeneouns silver impregnation of the neurons and excessive sample artifacts. The mercury-based neuron impregnation showed here, allowed us to clearly define whole neurons mainly because of a more continuous and homogeneous impregnation of the cells and much reduced density of artifacts (scattered reflexive granules) that interfere with image segmentation^30^. In addition, another advantage of the Golgi-Cox method is the increased probability of staining an increased number of different neuronal types than the Golgi method, what is of great interest for a large scale analysis of neuronal morphology in health and pathological conditions^31^.

For optimal visualization of the neuronal architecture, the raw data should be preprocessed to solve problems such as periodic noise and nonuniform brightness^32^. Then, the preprocessed data can be reconstructed and the volume rendered in 3D. Therefore, 2048 projections of each imaged structure were reconstructed. This number of projections, although does not fulfill the Crowther criterion, was the largest possible within the constraint of sample damage and mercury precipitation, as explained below.

The staining method used in this work involves the use of a photosensitive reaction based on the reduction of mercury ions into an insoluble salt. Typically, a standard histological procedure would follow a two-step process involving the impregnation of the tissue with an ionic mercury solution followed by a developing step with the formation of mercury sulfide upon an alkali treatment. Although not fully comprehended, the mercury concentration during the impregnation is not homogeneous but selective, prioritizing a small fraction of neurons followed by some glial cells^33^. However, because we were working with whole samples, the homogeneity of the alkali treatment throughout the sample could not be fully granted, and this second step was not done. In addition, this modification did not compromise the quality of the staining, as the X-rays would detect the absorbed mercury irrespective of its chemical state. This dynamic corroborates the observed darkening of the samples during acquisition, as the continuous X-ray exposition would reduce the mercury turning the sample darker as more mercury ions aggregate into denser salts (see Supplementary Fig. S1). In addition, since we are working with Golgi-Cox fixed samples, no X-ray radiation damage on tissue and cellular morphology was observed at this magnification, allowing the full reconstruction of the structures analyzed.

In a near future it would be tempting to map brain circuits in terms of connectivity between classes of different/same neuronal cell circuits (e.g. serotonergic, dopaminergic, etc.). However, such procedure is conceptually challenging and should combine technologies from different areas of science such as biology, chemistry, physics, engineering and nanotechnology in order to help us overcome these obstacles. Nonetheless, proper sample preparation is still a barrier common to all applied methods that still needs to be overcome. The investment in new methodologies that allow us to evaluate the brain-network in large structures, but do not require tissue sectioning, will provide a significant gain in time and resolution. Accordingly, the technique presented here opens the way for combining X-ray from synchrotron light source with new labeling technologies to cell type–specific and the use of trans-synaptic tracers, both with high specificity and great penetrance in large structures, paving the way for the visualization of cellular structure and connectivity in brain.

X-ray image reconstruction and segmentation has been greatly developed during the last few decades. Nowadays, with developments of new image reconstruction methods, it is possible to extend the field of view (FOV), keep the image resolution and make a full 3D image reconstruction in less than one minute, using graphic process units (GPU)^34^. In other words, images are becoming larger, and, reconstruction times, shorter. This fact transforms image segmentation into the bottleneck of this technique. To overcome this cutting edge challenge, image segmentation and analysis using high performance computing and machine learning are the most promising technique. In the field of brain connectomics, there is a noteworthy gap between the time required for data acquisition, which is extremely fast, and the reconstruction of the neural processes contained in the same dataset collected. As an example, to reconstruct *C. elegans* connectome it took thousand hours for data acquisition and around twelve years of part-time work by one scientist for segmentation^35^. In addition, the current methods do not present a total accuracy when segmenting complex samples in large datasets, therefore been insufficient for use without manual corrections. Data acquisition and reconstruction algorithms so far do not provide sufficient reconstruction accuracy of neural circuitry and both are extensively time-consuming^35^. Therefore, although imaging larger brain circuits is becoming each day more feasible, reconstructing the precise neuronal connection is still a problem. Currently, automated reconstructions generate erroneous neurite breaks or erroneous neurite mergers of neuronal path length, which results in the loss or misassignment of most of the connections of a given neuron^36,37^ (also, for example, see Fig. 4a,b, right panels). Correct sample preparation joining the creation of more accurate, automatic and fast segmentation tools are the keys to solve the challenges of modern connectomics.

In conclusion, we show that the technique presented here opens the way for 3D mapping and it is suitable for both synchrotron and table top tomograms. Moreover, the current developments and building of more sophisticated and powerful synchrotron light source, together with advancements in labeling and image processing software, will increase our spatial resolution while allowing the analyses of increasingly larger sample volumes at a nanometric resolution. Such line of research will play a pivotal role to understand the normal and pathological neural anatomy and network.

## Methods

### Animals Used

For this study, we used adult FVB/NJ (JAX#1800) mice. Animals were housed in the pathogen-free animal facility at the Brazilian Biosciences National Laboratory (LNBio) and maintained on a photoperiod of 12:12 light/dark cycle at 21–24 °C. Experimental protocols were carried out in strict accordance with the recommendations set forth in the Guide for the Care and Use of Laboratory Animals of the Brazilian National Council of Animal Experimentation (http://www.cobea.org.br/) and the Federal Law 11.794 (October 8, 2008). The Institutional Committee for Animal Ethics of the Brazilian Center for Research in Energy and Materials (CEUA-CNPEM, License 29-B) approved all the procedures used in this study. Effort was made to minimize the number of animals used and their suffering.

### Pilocarpine treatment

Mice were submitted to *Status Epilepticus* (SE) according to Cavalheiro, 1996^24^ and Araujo, 2014^38^. Briefly, animals (n = 15) received an intraperitoneal injection (i.p.) of atropine methyl nitrate (1 mg/kg in saline, Sigma, St. Louis, MO), 30 min before pilocarpine injection. SE was elicited with an intraperitoneal injection (i.p.) of pilocarpine (280 mg/kg in saline, Sigma, St. Louis, MO). SE was terminated after 6 h by diazepam (10 mg/kg, i.p.) to achieve uniform SE duration. Control animals (n = 5) were injected with atropine methyl nitrate (1 mg/kg) and then with saline, instead of pilocarpine.

### Golgi-Cox impregnation of samples for µCT

Fourteen days after SE, mice were euthanized using a CO_2_ chamber followed by decapitation. Brains were removed and then one hemisphere, cerebellum, hippocampus and frontal cortex were rapidly dissected, washed with distilled water and immersed in freshly prepared Golgi–Cox solution. Tissues were kept in this solution and protected from light for 14 days^39^. Fixation or perfusion with 4% PFA of brains were avoided since this leads to over-impregnated neurons and glial cells rendering segmentation of individual cells hard to perform^39,40^. After the incubation period, all samples were dehydrated in 70%, 80%, 95% and 100% ethanol and embedded in Paraplast Plus (Sigma Aldrich, St. Louis, MO). Figure 1a portrays the dissected areas of interest and tissue preparation procedure.

### Sample preparation for histology

Brains (n = 3 control group; n = 3 pilocarpine group) were incubated in Golgi-Cox staining solution for 14 days, protected from light, as previously described^39^. After the incubation period, brains were serially sectioned (150 µm) using a vibratome (VT 1000 S Leica, Germany). Brain slices were placed in gelatin-treated slides, air dried at room temperature, rinsed in 28% ammonia solution for 30 min and washed twice (5 min each) in distilled water. Subsequently, the sections were immersed in fixer solution (15%) in distilled water for 15 min, washed twice in distilled water for 5 min and then dehydrated in a series of graded ethanol concentration (5 min each, 70%, 80%, 95% and 100%), cleared in xilol and mounted in Entellan mounting media (Merck, Darmstadt, Germany).

### IMX Beamline Setup (UVX-LNLS)

The stained Paraplast embedded samples are self-sustainable and were mounted on a stub at the rotation stage. More than 2000 X-ray transmission images were acquired by revolving the sample around a fixed rotation axis by 360 degrees, in uniformly spaced angular steps, to produce a stack of sinograms (Fig. 2a) that were later computationally transformed into a 3D map of the electron density of the sample.

The transmission images of the whole brain and dissected brain regions were obtained using radiation from the 1.67 T bending magnet of the 1.37 GeV UVX storage ring, filtered by 0.9 or 0.2 mm Si filters, respectively. These setups produced a polychromatic beam, with peak energy at approximately 15 keV or 11 keV, respectively, and approximately 50% bandwidth. The radiographs were recorded by an indirect detector system, based on a thin scintillator that converts the transmitted X-rays into visible light. The light is focused on a PCO2000 CCD sensor by an infinity corrected optics, which can produce an adjustable magnification of the visible light image radiating from the scintillator, yielding X-ray projection images with equivalent pixel sizes of 0.82 × 0.82 μm^2^ or 4.11 × 4.11 μm^2^. Considering the array size of 2048 × 2048 of the PCO2000 CCD, the field of view (FOV) of the radiographs were 1.7 mm × 1.7 mm for the high-resolution images; and 8.4 mm × 8.4 mm for the low-resolution images. In order to increase the FOV to image larger samples, the rotation axis was positioned near to the edge of the FOV and an acquisition covering an angular range of 360 degrees in uniformly spaced angular steps was used to compose a complete sonogram (Fig. 2a). This increased the effective detector pixel number up to 3072, as well as the 3D image size from 8.5Gpx (2048^3) to 19.4Gpx (3072^2*2048)). A final FOV of 12 mm, for the low-resolution images, was achieved, which was suitable for imaging a whole mouse brain. Also, for single cells imaging, the final FOV was of 2.4 mm. Therefore, it was necessary to perform the dissection of the brain structures into smaller pieces of tissue so it could fit in front of the FOV. On both high and low resolution, a total of 2048 projections and exposure time of 1 second per projection were used to compose the necessary dataset for image reconstruction using the BST approach^41,42^. All the data acquisition conditions are summarized in Table 1.

**Table 1:** Parameters for data acquisition condition.

Instrumentation	Skyscan 1272	IMX
X-ray energy	45 (kV) Al 0.5 + Cu 0.038 filters	Pinkbeam (11 keV mean)
Pixel size (μm)	1.0	0.82
Viewing field (mm)	2.4	2.4
Rotation/frame (degree)	0.2	0.17
Exposure/frame (ms)	8793	1000
Frame/data set	2415 × 2415	2048 × 2048
Data set acquisition time (min)	190	40

### Setup Bench μCT

Paraplast embedded samples (n = 3 hippocampi of each group) were scanned using a Brucker Skyscan 1272 μCT scanner (Bruker, Brussels, Belgium). Total acquisition time of 190 min was used for a spatial resolution of 2 µm and a pixel size of 1 µm. The exposure time was fixed at 8.79 s. The voltage was 45 kV, and the rotation step 0.20°, with Al 0.5 μm + Cu 0.38 μm filters. The projection data were corrected for distortion and reconstructed by adjusting, smoothing and correction of ring artifact and beam hardening. The reconstructed isotropic voxel size, after reconstruction, was 2 µm. 8-bit images were reconstructed with the scanner software (NRecon 1.6.6.0, Skyscan, Brucker μCT, Belgium). All the data acquisition conditions are summarized in Table 1.

### Data processing

Image quantification and some specific visualization is only possible from segmented images, *i.e*., labeled image, where each voxel of the image is correlated to one region of your sample, *e.g*. cell body, neurons, etc.

For that, the 3D reconstructed data obtained at the IMX beamline was post processed using Avizo Fire 9.4, a 3D commercial visualization software (https://www.fei.com/software/avizo-for-materials-science/). A non-local means filter was applied to all images to remove noise and enable the image segmentation and quantification. Different methodologies of data analysis were applied, depending on the brain region and type of analysis and quantification:

1. Whole brain segmentation (Fig. 2b): Watershed tool was used to separate the brain region from the background, based on seeds created by a single threshold tool. The 3D volume was created using the isosurface rendering tool. No quantitative analysis was made for this image.
2. Cell body segmentation (bright spots of Fig. 4a): A single threshold followed by a dilation operation, which selects and grows the voxel region, was used to select the cell body regions. Based on this segmentation, we quantify the amount of cell body just by counting the number of individual elements larger than 10 μm in diameter.
3. Neurons segmentation (Fig. 5b): an iterative segmentation was required due to the high complexity of these structures. First, a combination of single threshold, dilation and erosion were applied, followed by a manual correction of unselected voxels, using a brush tool. Spurious small regions that were still labeled as neurons were removed using the ‘remove island’ tool.

For a comprehensive review of image analysis, see ref.^43^. All the parameters for image segmentation are summarized in Table 2. It is important to highlight that the segmentation methodology detailed in this manuscript can be applied to any other type of image, including two-dimensional. However, the threshold values depend on the reconstruction algorithm and some adjustments might be necessary. 8-bit images were reconstructed with the scanner software (NRecon 1.6.6.0, Skyscan, Brucker μCT, Belgium). 3D visualization of scanned objects by volume rendering was performed with the CTVox program (Skyscan, Brucker μCT, Belgium).

**Table 2:** Parameters for image segmentation.

	Gray level	Selected region/seeds	Purpose
Whole brain	130 to 256	Brain seeds	Volume rendering
	0 to 126	Background seeds	
Cell body	142–255	Bright Spots selection larger than 9 µm in diameter	Cell number quantification
Neuron	139–255	Neuron body manual selection	Manual 3D morphological tracing

### Statistics

Quantitative results of Fig. 7 are expressed as mean values ± S.D. Prism 6.0 (GraphPad, La Jolla, CA) was used for data analysis. Groups of data were compared using Student’s t test, and p value < 0.05 was taken to indicate statistical significance.

### Ethical approval and informed consent

Experiments carried out with the animals were in strict accordance with the recommendations set forth in the Guide for the Care and Use of Laboratory Animals of the Brazilian National Council of Animal Experimentation (http://www.cobea.org.br/) and the Federal Law 11.794 (October 8, 2008). The Institutional Committee for Animal Ethics of the Brazilian Center for Research in Energy and Materials (CEUA-CNPEM, License 29-B) approved all the procedures used in this study.

## Electronic supplementary material

Supplementary Information

Supplementary Movie S1

Supplementary Movie S2

## Data Availability

All data generated or analyzed during this study are included in this published article (and its Supplementary Information files).
