# Supplementary material for: High-resolution synchrotron-based X-ray microtomography as a tool to unveil the three-dimensional neuronal architecture of the brain
Source: Sci Rep. 2018 Aug 13;8:12074. doi: 10.1038/s41598-018-30501-x (PMC6089932; doi:10.1038/s41598-018-30501-x)
Supplement: Supplementary file 1 — Supplementary Information [file 41598_2018_30501_MOESM1_ESM.pdf]

High-resolution synchrotron-based X-ray microtomography as a tool to unveil the three-dimensional neuronal architecture of the brain

Matheus de Castro Fonseca[1]*; Bruno Henrique Silva Araujo[1]; Carlos Sato Baraldi Dias[2]; Nathaly Lopes Archilha[2]; Dionísio Pedro Amorim Neto[1]; Esper Cavalheiro[3]; Harry Westfahl Jr[2]; Antônio José Roque da Silva[2]; Kleber Gomes Franchini[1].

***corresponding author**

[1]Brazilian Biosciences National Laboratory (LNBio), Brazilian Center for Research in Energy and Materials (CNPEM), Zip Code 13083-970, Campinas, Sao Paulo, Brazil

[2]Brazilian Synchrotron Light National Laboratory (LNLS), Brazilian Center for Research in Energy and Materials (CNPEM), Zip Code 13083-970, Campinas, Sao Paulo, Brazil.

[3]Department of Neurology and Neurosurgery, Federal University of São Paulo (UNIFESP/EPM), Zip Code 04021-001, São Paulo, São Paulo, Brazil

Corresponding author: * Matheus de Castro Fonseca, Ph.D.

Brazilian Biosciences National Laboratory (LNBio),

Brazilian Center for Research in Energy and Materials (CNPEM),

Giuseppe Maximo Scolffaro Av. Campinas, SP- Zip Code 13083-970 – Brazil,

Phone: (19) 35121119. E-mail: matheus.fonseca@lnbio.cnpem.br

Supplementary Figure S1

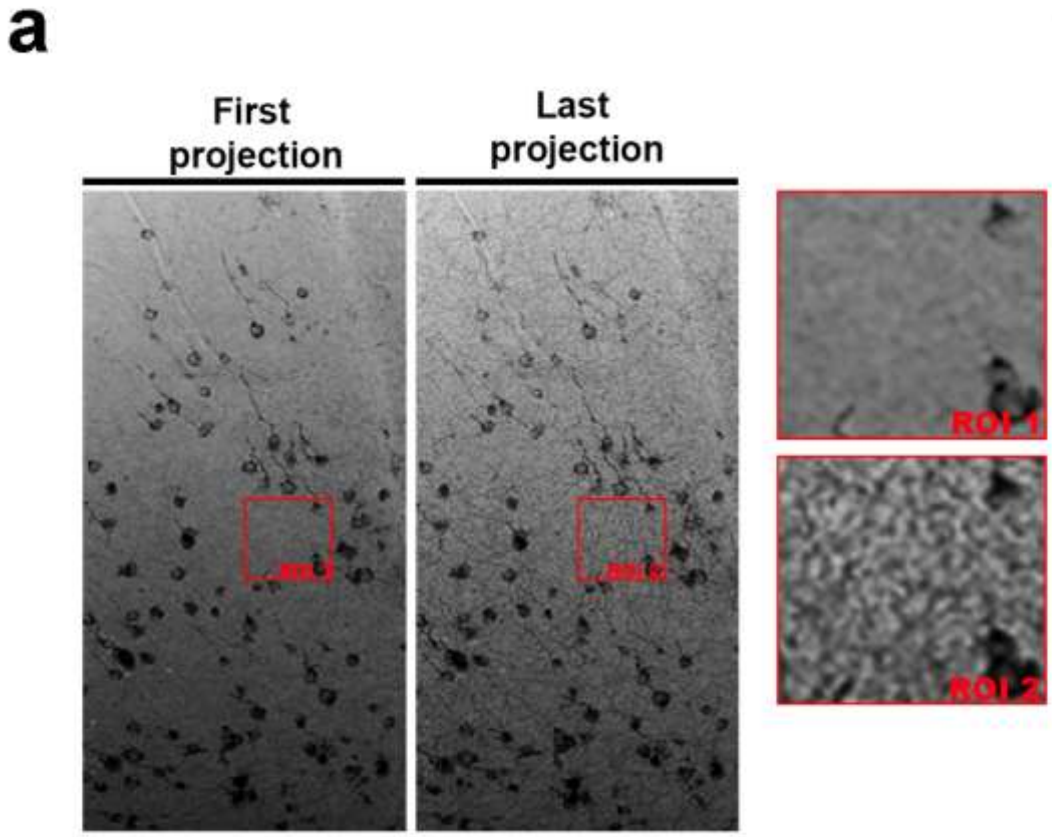

Supplementary Fig. S1 legend. First and last projection from a 2048 acquisition set of a mercury-impregnated control frontal cortex. **(a)** The selected ROIs show aggregation of mercury salts. This homogeneous revelation of the ionic mercury occurs throughout the sample, including the neurons. Although this event contributes to an increased background, it did not cause any harm to the reconstruction of independent neurons.

Supplementary Movies Legends

Supplementary Movie S1. A visualization of an isolated mercury-impregnated cortical neuron while scrolling through the virtual z-plane of the reconstructed image. This video is composed of 246 slices (around 202 µm in depth) and allows the full tracing of the cell and its reconstruction in 3D, as shown on Movie S2.

Supplementary Movie S2. A 3D reconstruction of the neuron sliced in Supplementary Movie S1. This video represents the 3D reconstruction of a 246-virtual sliced image. Observe that this process allows the segmentation of the whole cell without mistracing of the neurites.
